# Dentists' preparedness to provide Level 2 services in the North East of England: a mixed methods study

**DOI:** 10.1038/s41415-023-5569-3

**Published:** 2023-03-07

**Authors:** Sarah Simpson, Christopher K. Wallace, Malcolm Smith, Paul Blaylock, Gillian Vance

**Affiliations:** 41415160328001 Health Education England, North East and North Cumbria, UK; School of Dental Sciences, Newcastle University, UK; 41415160328002 School of Dental Sciences, Newcastle University, UK; 41415160328003 Health Education England, North East and North Cumbria, UK; 41415160328004 School of Medical Education, Newcastle University, UK

## Abstract

**Background ** Dentists capable of delivering Level 2 complexity treatments may benefit patient access to dental care and increase workforce morale. However, little is known about attitudes, capability and training needs relating to Level 2 dental services.

**Aims ** To examine attitudes to, and perceived readiness for delivery of Level 2 dental services by NHS dentists in the North East of England and North Cumbria.

**Method ** A sequential mixed methods study with an online survey and semi-structured interviews. Participants were general, community, and hospital-based dental practitioners. Descriptive statistics of survey and thematic analysis of qualitative data were undertaken.

**Results ** Overall, 56% of 124 respondents had a limited understanding of the Level 2 performer role. A minority felt they were already providing Level 2 care across all speciality areas; 9% (n = 11) had a professional portfolio suitable for Level 2 accreditation. Confidence to undertake Level 2 competencies varied by speciality area, being highest in paediatric dentistry, and lowest in endodontics and orthodontics. Qualitative data identified motivations, and personal, organisational and system factors that were seen as barriers or enablers to upskilling.

**Conclusion ** The regional NHS workforce is unprepared to provide Level 2 dental services. Successful introduction will require review of necessary infrastructure and transparency of accreditation and contracting processes.

**Supplementary Information:**

Zusatzmaterial online: Zu diesem Beitrag sind unter 10.1038/s41415-023-5569-3 für autorisierte Leser zusätzliche Dateien abrufbar.

## Key points

- Provides insight into workforce self-reported readiness to provide NHS commissioned Level 2 dental services.
- Highlights factors which may limit the uptake of Level 2 training and delivery.
- Outlines interest in, and expectations of, education and training for Level 2 skill development.

**Supplementary Information:**

Zusatzmaterial online: Zu diesem Beitrag sind unter 10.1038/s41415-023-5569-3 für autorisierte Leser zusätzliche Dateien abrufbar.

## Introduction

NHS dentistry is in crisis. There is a backlog of unmet patient need caused by service disruptions during the COVID-19 pandemic and workforce morale is at an all-time low.^1^^,^^2^ Increasing numbers of primary care dentists are leaving the NHS or the profession entirely, which, in turn, exacerbates workload pressures.^3^^,^^4^

Currently, approximately 12% of the dental workforce hold specialist titles and the specialist workforce is disproportionately situated across the UK.^5^^,^^6^^,^^7^ Patients requiring specialist services therefore face limited and inequitable access.^7^^,^^8^^,^^9^ Policymakers are now faced with the monumental challenge of leading change that will address this care crisis.

One option proposed to address these problems in England is to develop a workforce who can provide NHS commissioned 'Level 2 dental services'. Individuals within this workforce are termed 'Level 2 performers': dentists with enhanced skills, who may or may not be a specialist, and can competently provide dental care of enhanced complexity (Level 2), intermediate to that expected of general dental practitioners (GDPs) and specialists or consultants.^10^

Evolving from the historical concept of 'dentists with special interests', it is proposed that the Level 2 workforce would comprise of specialists and GDPs who have undertaken further training.^11^^,^^12^ This 'upskilling' of GDPs could help lessen the perceived divide between primary and secondary care, allowing patients to access an appropriately skilled member of the dental workforce closer to home and so help reduce oral health inequalities.^10^^,^^13^ Further, enabling GDPs to carry out more complex dental services may improve job satisfaction and thereby promote a sustainable workforce in NHS dentistry.^8^

Level 2 services can be provided in eight speciality areas: endodontics; periodontology; prosthodontics; oral surgery; special care dentistry; paediatric dentistry; orthodontics; and sedation.^14^^,^^15^^,^^16^^,^^17^^,^^18^^,^^19^ Commissioning standards or 'guides' have been published in each of these speciality areas, setting out expectations for commissioned services, treatment complexity levels and a set of 'mandatory clinical competencies' that Level 2 performers would be expected to provide.^13^

Competency can be gained through speciality training, Level 2 training programmes, or alternative routes where evidence of competency achievement is required. Access to Level 2 training and consistency of opportunities, however, varies considerably across England.^20^^,^^21^^,^^22^

Once trained, dentists need to be both accredited as a Level 2 performer and awarded an NHS Level 2 contract. Performer accreditation is awarded by local panels who assess an individual's professional portfolio.^22^ Portfolios should include evidence of Level 2 competency achievement, feedback from referrers and patients, and references from two consultants or specialists.^22^ Organisations with accredited performers can seek a Level 2 contract through the NHS commissioning procurement processes.

There has been some early exploration of the development of the Level 2 workforce in restorative and paediatric dentistry.^23^^,^^24^ Published evaluations of upskilling initiatives have demonstrated high patient satisfaction with services provided by primary care dentists with enhanced skills and the majority of dentists involved in the initiatives recognised patient benefits.^12^ However, the willingness, capacity and training needs of the dental workforce to deliver Level 2 services are unknown.

This study aims to add to the dental literature by examining attitudes to, and perceived capability and training needs for, delivery of Level 2 dental services by NHS dentists in one area of England - the North East of England and North Cumbria (NENC).

## Materials and methods

The study adopted a sequential mixed methods design involving data collection from dentists in general dental services (GDS), community dental services (CDS) and hospital dental services (HDS) in NENC. The region has approximately 424 NHS general dental practices, six multi-site community dental services and one hospital dental service.^25^

### Phase 1

A self-completed, anonymous, online survey was distributed to practising GDC-registered dentists in NENC. Specialists and consultants were excluded. It was not possible to gain accurate regional workforce figures. However, NHS Business Services Authority and Health Education England (HEE) working across NENC data were used to estimate a population size of 2,150 dentists.^25^

#### Survey design

The 65-item survey was adapted from a previously published survey and further developed to incorporate the Level 2 commissioning standards and perspectives of a project panel of 13 dental workforce stakeholders (including GDPs, community dental officers, trust grade dentists, dental core trainees, specialists and consultants).^23^^,^^26^

Section one collected data on respondent' demographics, including sex, qualifications, job role and workplace.

Section two explored respondents' experience of, and confidence in, undertaking clinical practice in Level 2 commissioned speciality areas. Closed questions were used to establish self-perceived ability to provide Level 2 services, interest in obtaining a Level 2 contract and undertaking relevant training in each speciality area.

A Likert Scale was used to explore the frequency with which clinical practice in each speciality area was undertaken (never, daily, weekly, monthly, quarterly, less than quarterly). A Likert Scale was also used to establish respondents' self-perceived confidence across 32 Level 2 speciality specific competencies that had been identified by the project panel as essential for a Level 2 performer (very confident, confident, neutral, somewhat lacking confidence, severely lacking confidence).^14^^,^^15^^,^^16^^,^^17^^,^^18^^,^^19^

In section three, closed questions were used to ask respondents if they had a professional portfolio suitable for a Level 2 accreditation application. Section four invited free-text responses about operating as a Level 2 performer.

Amendments were made following piloting with nine representatives from GDS, CDS and HDS to ensure face and content validity.

#### Survey distribution and data collection

The survey was conducted using online survey software (Online Surveys, Jisc, Bristol, UK). A voluntary response sampling frame was adopted. It was distributed using a range of regional dental networks (Local Dental Chairs, HEE NENC, regional membership of Northern Dental Practice Based Research Network, Faculty of General Dental Practitioners, British Dental Association) and social media. The survey was open for ten weeks in total (3 March 2021 - 12 May 2021) with reminder emails and social media posts released at two and four weeks.

#### Quantitative data analysis

Descriptive statistical analysis was undertaken. Responses 'very confident' and 'confident' are grouped as 'confident', while 'somewhat lacking confidence' and 'severely lacking confidence' are grouped as 'unconfident' for analysis. Data were dichotomised into two groups based on year of graduation - those who graduated before and after 2010 (median year of graduation) - for cross tabulation analysis. Chi-squared tests were used to determine statistically significant differences between the two groups where p <0.05.

### Phase 2

#### Sampling and procedure

Participants were selected from those who completed the online survey and through a snowballing technique to ensure workforce groups of interest (GDS, CDS, HDS) and genders were represented.

Semi-structured interviews were undertaken using remote video link by one researcher (SS). These followed a topic guide developed from survey findings. All interviews were audio-recorded, transcribed verbatim using Microsoft Teams (version 1.5.00.8070) and manually checked (SS).

#### Qualitative data analysis

Data from free-text survey responses were analysed using an inductive process in which raw data were read and coded independently using Nvivo 12 Pro (QSR) by SS and CW (speciality trainees in paediatric dentistry) for a proportion of responses. Following consensus discussions (SS and CW), a coding framework was created and applied to the remaining data. Frequency of code occurrence and underlying context were considered while performing a content analysis on the full dataset. Initial themes were developed independently (SS and CW) and refined during discussions until agreement on final themes was reached.

Semi-structured interview transcripts were analysed using reflexive thematic analysis.^27^ Three transcripts were reviewed by SS and CW to identify initial codes. These were then discussed among researchers (SS, CW and GV) to allow interpretation of codes and definitions to be refined and agreed. All remaining transcripts were coded independently by SS and sorted codes were reviewed by researchers (SS; GV) to develop descriptive themes through a series of iterations.^28^

Ethical approval was granted by Newcastle University Ethics Committee (Ref: 9181/2020). Written consent was obtained from all participants.

## Results

### Quantitative data (phase 1)

#### Demographics

**Fig. 1:**
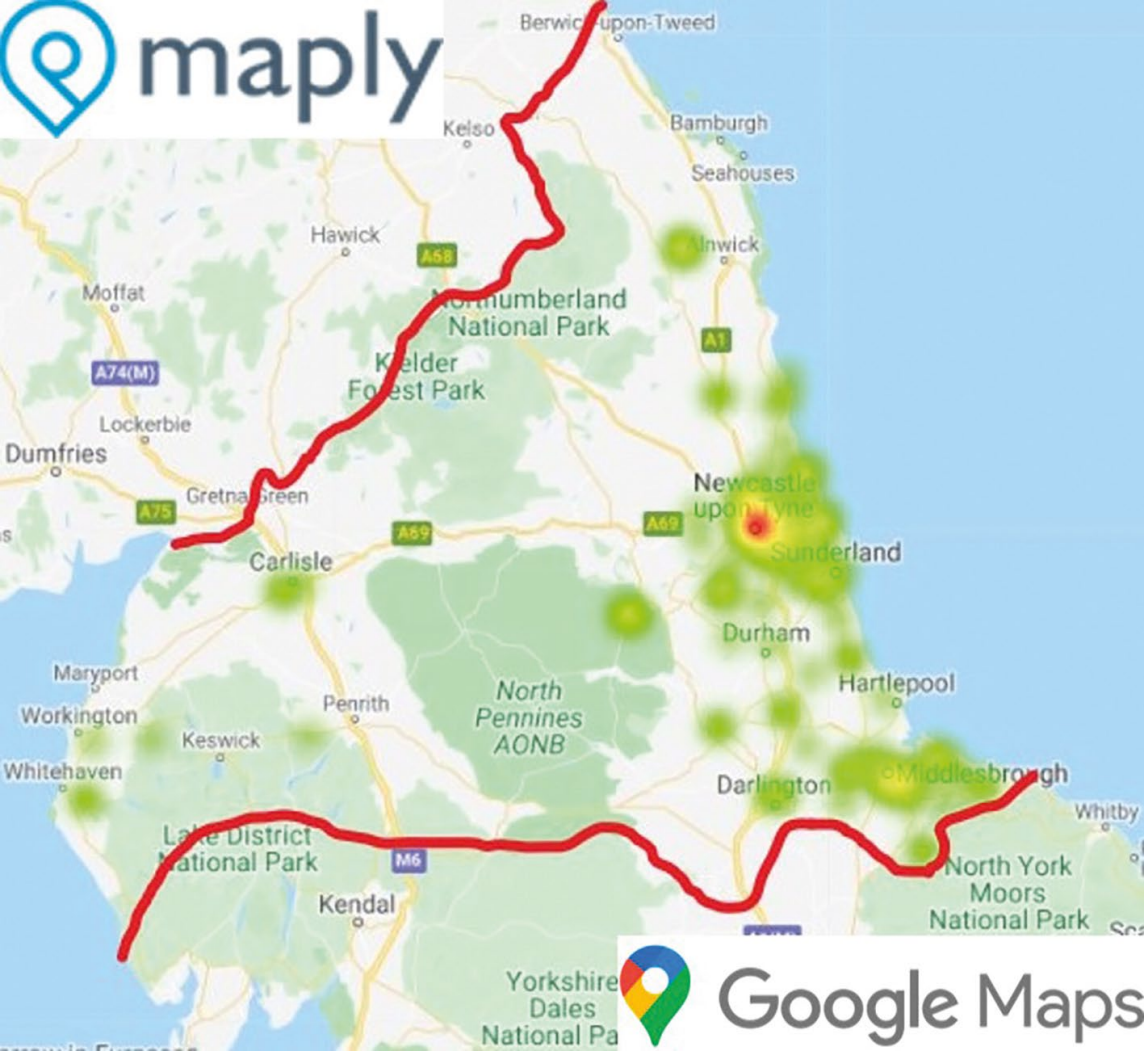
Heat map illustrating respondent workplace postcode. Plotted using Maply (https://maply.com); map data 2021 Google Maps

In total, 124 respondents met the inclusion criteria for analysis. Just over half were women (56%; n = 70), 43% men (n = 53) and the remaining non-binary (n = 1). Most respondents (63%) were GDS dentists (n = 78). Respondent job roles are shown in Table 1. Respondent year of graduation ranged from 1980-2020, with the median year of graduation being 2010. Figure 1 illustrates respondents' primary workplace postcode.

**Table 1:** Demographic data of respondents in quantitative phase (dental service and teaching commitments responses were not mutually exclusive, therefore number of respondents equate to greater than n = 124)

Demographic data	Number of respondents (n = 124)	Proportion of respondents (%)
**Job role**		
Associate dentist	48	39
Principal dentist	30	24
Dental core trainee (DCT1, DCT2, DCT3)	12	10
Foundation dentist (FD)	9	7
General professional trainee (longitudinal FD and DCT1)	8	6
Senior dental officer	10	8
Dental officer	3	2
Other	4	4
**Respondent service contribution**		
NHS treatment in general dental practice (at least one session/week)	92	74
Private treatment in general dental practice (at least one session/week)	50	40
Teaching/clinical supervision	29	23
Hospital dental services	24	19
Community dental services	17	14
Out of hours dental care	9	7
**Number of years of post-graduate training experience**		
0	8	6
1	69	56
2	33	27
≥3	14	11

The majority (74%) were involved in the delivery of NHS treatment within GDS. Respondents' service contribution and total years of experience of formal post-graduate training programmes are shown in Table 1. The majority had completed dental foundation training (n = 83) and general professional training (longitudinal FD-DCT1; n = 25). Most (68%; n = 84) had completed at least one post-graduate qualification, particularly Membership of the Faculty of Dental Surgery or Membership of the Joint Dental Faculties (n = 55).

#### Overall perception

Over half of respondents (56%; n = 70) reported that they did not have a clear understanding of the Level 2 performer role.

As shown in Table 2, a minority felt they were currently working at the level of a Level 2 performer in each speciality area. Some, however, reported to be commissioned to deliver Level 2 services and a few reported to be both accredited and commissioned to deliver Level 2 services.

**Table 2:** Respondent self-perception of currently working at the level of a Level 2 performer, being commissioned and being both accredited and commissioned as a Level 2 performer in each speciality area (note: commissioned refers to an individual being awarded a Level 2 NHS contract and accredited refers to individuals who have been recognised by a local accreditation panel as being competent to deliver Level 2 care)

Speciality	Currently working at the level of a Level 2 performer				Commissioned as a Level 2 performer				Accredited and commissioned as a Level 2 performer			
	Yes		No		Yes		No		Yes		No	
	n	%	n	%	n	%	n	%	n	%	N	%
Oral surgery	19	15	105	85	2	2	122	98	1	1	123	99
Sedation	15	12	109	88	27	22	97	78	6	5	118	95
Special care dentistry	14	11	110	89	10	8	114	92	1	1	123	99
Paediatric dentistry	13	10	111	90	9	7	115	93	1	1	123	99
Endodontics	13	10	111	90	0	0	124	100	0	0	124	100
Periodontology	9	7	115	93	0	0	124	100	0	0	124	100
Prosthodontics	8	6	116	94	0	0	124	100	0	0	124	100
Orthodontics	5	4	119	96	4	3	120	97	1	1	123	99

#### Interest and confidence

**Fig. 2:**
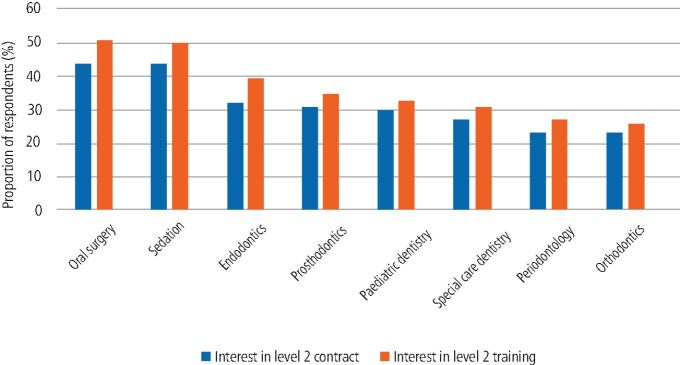
Respondent interest in gaining a Level 2 contract and undertaking Level 2 training in each speciality area

Interest in gaining a Level 2 contract varied from 23-44% (n = 29-55) and to undertake training from 26-51% (n = 32-63), depending on speciality area (Fig. 2). Most popular were oral surgery (interest in contract: 44%, n = 55; interest in Level 2 training: 51%, n = 63) and sedation (44%; n = 55 and 50%; n = 62, respectively).

Confidence varied by speciality and by competency within each speciality, as shown in online Supplementary Table 1. Confidence was highest in paediatric dentistry with between 41% (n = 51) and 45% (n = 56) of respondents self-reporting as confident depending on the specific competency. Respondents were least confident in orthodontics with between 69% (n = 86) and 80% (n = 99) unconfident, depending on the specific competency.

Cross tabulation demonstrated pre-2010 graduates were generally more confident than those graduating post 2010 in endodontics, prosthodontics and orthodontics. Significant (p <0.05) differences in confidence were found for several competencies as shown in online Supplementary Table 1. Self-reported confidence in the full range of Level 2 competencies is provided in online Supplementary Table 2.

#### Professional portfolio suitable for Level 2 accreditation

Only 9% of respondents (n = 11) reported having a portfolio that would fully meet accreditation requirements. Just under half reported that their portfolio would partially meet (44%; n = 54), or meet none, (47%; n = 59) of the requirements. Items most frequently absent from portfolios were references from two consultants/specialists (69%; n = 37) and evidence of feedback from patients and referrers (61%; n = 33).

### Qualitative data (phase 2)

**Fig. 3:**
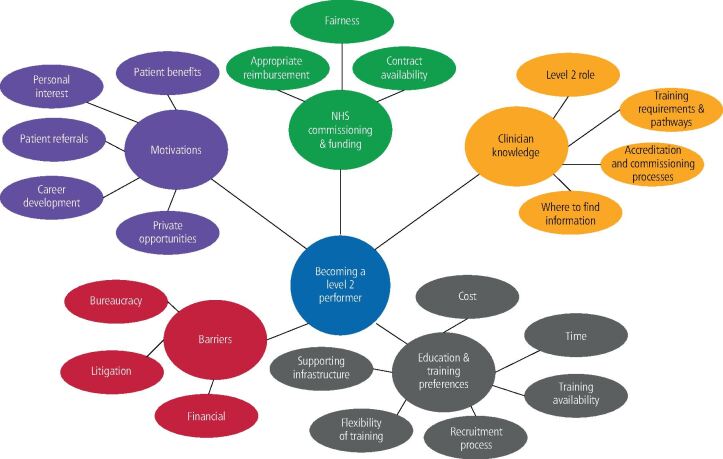
Diagram of themes and subthemes generated from respondent views on becoming a Level 2 performer

Qualitative data from survey free-text and interviews were analysed separately. Findings from both are illustrated in Figure 3.

#### Free-text responses

There were 224 free-text responses giving views on Level 2 service delivery and training. Four main themes were produced.

#### Clinician knowledge

Most often, responses indicated a lack of knowledge around the Level 2 performer role and how to access information about becoming trained and accredited:

- '*Don't know very much about it and wouldn't know how to go about becoming one*' (FD, graduated 2019).

#### NHS England commissioning and funding arrangements

Many expressed disillusionments about the current contractual arrangements, which meant that they doubted reimbursement for Level 2 services would be adequate, or reflect the increased skill, equipment, materials and time required. Further, Level 2 NHS contracts were seen as inaccessible, leading to a feeling of futility, with some questioning feasibility of Level 2 services:

- '*When I have previously looked at applying for Level 2, the payment for completing complex procedures made these, more difficult treatments, less profitable per hour than the simple treatments I am currently providing. I would be taking on the treatments, and their associated risk for the joy of doing dentistry*' (GDS dentist, graduated 1991)
- '*Happy to undertake, but not happy to pay significant cost if no guaranteed [Level 2] job/funding at end of training*' (HDS, graduated 2016).

#### 'Costs' of training and accreditation

In addition to financial implications of undertaking training, respondents noted a tension between balancing existing work commitments and personal life.

Perceptions of competitive recruitment processes for training programmes could dissuade applicants, while flexibility and opportunities for local training were important factors influencing willingness to train. Furthermore, the accreditation process, and especially meeting portfolio requirements, was thought to be prohibitive:

- '*I feel that there are many well-experienced practitioners working at Level 2 who would not be able to jump through [accreditation] hoops who should be considered*' (CDS, graduated 2009).

#### Opportunities and threats

Personal benefits were identified by many, including adding interest and variety to their case load. There was acknowledgement that these skills could improve job satisfaction and workforce retention. Benefits to patients were noted, with increased access and improved quality of care:

- '*It would help to retain dentists in the profession by maintaining their interest'* (GDS, graduated 2001).

There were some negative views relating to perceived bureaucracy. Respondents felt processes around accreditation and contracting were likely to be unnecessarily complex.

#### Interviews

Participants had 2-30 years of practising experience and included three GDS, three CDS, two HDS and two DCT dentists. Most participants were women (n = 7).

Three major themes were identified:

- Motivations to undertake Level 2 training
- Education and training preferences
- Barriers and enablers to upskilling.

#### Motivations

Participants described a range of personal, organisational and patient benefits to becoming a Level 2 performer. These mirrored those cited in free-text and included the opportunity to expand the range and quality of speciality services provided within GDS, increasing patient flow and scope for private growth:

- '*I'd prefer to have specialists or people with special interests in my practice rather than referring them to the hospital or other practices. I think also, financially, it's better for us if we're not sending patients away from the practice'* (GDS, graduated 2005).

While all participants acknowledged benefits of Level 2 accreditation for patient care, dentists working in community and hospital services raised the importance of benefits to individual practitioners, while GDS dentists cited organisational factors:

- '*Anything that involves keeping yourself current and up to date is really important for yourself and your job satisfaction'* (CDS, graduated 2014)
- *'If you're asking me as a dentist, it [Level 2] is not something I've ever really thought to do. If you're asking whether I would consider it within the practice…then probably yes'* (GDS, graduated 2005).

This suggests that an individual's attitudes to career development may be influenced by their current job role.

#### Preferences for education and training

Participants valued a mixture of didactic and hands-on flexible training. Supervised clinical practice and formal assessment of competence were the preferred modes of delivery and assessment.

Participants were open to both self-funded and hybrid funding models, with NHS England and HEE being expected to contribute to funding. In the hybrid model, some participants felt that learners should be expected to make a service commitment in return for funding. Such a commitment was seen variously as being stressful and a deterrent, as well as offering welcome stability:

- '*If you pay for the sedation diploma and then you might not have a sedation job afterwards and you forked out X amount for it, I think that's far less attractive than saying, "listen, we're going to give you the Level 2 [contract] and you're tied-in for X amount". At least you've got the security of knowing you've got the contract'* (HDS, graduated 2017).

#### Barriers and enablers to upskilling

Participants described a range of factors influencing the likelihood of pursuing Level 2 training.

#### Personal

Echoing free-text findings, Level 2 services and opportunities for career development were poorly understood by interview participants, suggesting a need for awareness-raising.

Current level of experience was an important factor. Those in their early careers often felt that they were too inexperienced to pursue Level 2 training. On the other hand, more experienced dentists felt that their level of experience negated the need to undertake further training. This suggests that awareness-raising needs to take place at the earliest (undergraduate) stages so that this option becomes integrated into UK training and career pathways.

Ongoing access to support systems was also an important factor, as it was felt to influence clinician confidence in Level 2 practice and reduce fear of litigation:

- '*If there was a community of practitioners carrying out Level 2 care that got together and if you didn't feel like you were on your own. If there was maybe "meet ups" where you could discuss cases with other clinicians, that would be a good thing'* (HDS, graduated 2015).

#### Organisation

Participants felt that the infrastructure of their organisation and regional dental services was crucial to realising Level 2 services. Undertaking Level 2 education and training was seen to be futile if there were no financial benefits or mechanism to provide NHS commissioned services.

There was a need to ensure that Level 2 performers have access to appropriate numbers and complexity of cases to gain and maintain competence, meet accreditation requirements and ensure they can fulfil contractual obligations.

Furthermore, some participants highlighted the importance of 'supply' and 'demand': proximity to the regional dental hospital, number of existing Level 2 service providers and patient demand for these services were all seen as determining viability of Level 2 provision:

- '*I guess it's quite niche and if we've already got other providers on our doorstep, will there be such a dilution of referrals that no one's going to be able to meet the targets?*' (GDS, graduated 1998).

#### System

Many participants regarded the requirements for accreditation as being reasonable but some highlighted issues of inequality and bureaucracy in the process. They felt dentists with secondary care experience would be able to fulfil accreditation requirements more easily than those based in primary care.

Some participants raised concern that there was insufficient workforce capacity to provide Level 2 training and delivery, as well as maintain Level 1 and 3 service provision. Consequently, this would have a knock-on effect for accessibility and timeliness of patient care. They raised the need for key policy stakeholders, such as HEE, to have strategic oversight of the sustainability of the Level 2 workforce pipeline.

Some acknowledged that Level 2 services gave opportunities for growth in private practice, which represented a risk to the NHS workforce. The likelihood of migration to the private sector was felt to be increased by costs of training, a lack of Level 2 NHS contracts and perceptions of inadequate NHS remuneration:

- '*Potentially, you've got a very well-trained individual who then goes off and says "right I'm doing private only now". I dare say, it's probably not something that could be done or policed for that matter, but it would be a shame for potentially the NHS or the taxpayer to be funding something like that'* (GDS, graduated 1998).

## Discussion

These data suggest that overall awareness of the processes around Level 2 training, accreditation and commissioning is limited. Although interest in training and contracts was high, confidence in performing Level 2 competencies and readiness for the accreditation process was generally low. Particular challenges were noted in endodontics and orthodontics.

Confusion about Level 2 processes were evident, given that some respondents reported that they were accredited and commissioned, despite, to our knowledge, there being no accredited and few commissioned performers in the region at the time of data collection.^29^ One explanation could be confusion over the varying terminology used, for example, a 'Level 2' or 'Tier 2' dentist, or a 'dentist with special interests'. Clarity around role and process expectations will be critical to successful integration of Level 2 services into the landscape of NHS dentistry.

Confidence was most commonly reported for paediatric dentistry competencies. This could reflect the differing nature of paediatric dentistry competencies. For example, endodontics involves very technical procedures, such as 'anatomical challenges such as root canal curvature >30 °', whereas paediatric dentistry involves cognitive and treatment planning competencies, such as 'managing patients where behavioural/psychological development, significant anxiety, medical co-morbidity, or disability increase complexity of care'. Lowest confidence was reported in orthodontic and endodontic competencies, which may highlight workforce training needs. Identification of workforce training needs are instrumental in guiding the development of Level 2 training programmes.

The quantitative data suggest that only a small proportion of respondents had a professional portfolio suitable for accreditation. This was supported by the qualitative data which indicated that dentists feel the accreditation process is more accessible to those working in a secondary care setting. It was, in effect, a vicious cycle whereby a portfolio cannot be developed without access to Level 2 complexity patients and consultant/specialist supervision, but these cannot be accessed without either being Level 2 accredited, or in a Level 2 training programme. And, in turn, these cannot be accessed without a suitable professional portfolio.

At least 23% of respondents were interested in Level 2 training and contracts in at least one speciality area. Respondents anticipated that Level 2 practice could benefit organisations and individuals by fulfilling personal interest and broadening practice. This may have a positive impact on job satisfaction and workforce retention.

### Comparison to existing literature

High levels of confidence in paediatric dentistry have been reported in a similar study which explored confidence in a range of Level 1 and 2 competencies. In that study, 44% of respondents expressed interest in providing Level 2 paediatric dental services compared to 30% in this work.^23^ Higher levels of interest may have been due to the survey content of the earlier study, which solely focused on paediatric dentistry, meaning respondents may have been a self-selected group with existing interest in this speciality. Nonetheless, the notably higher levels of confidence and interest in paediatrics make it an ideal starting point to optimise Level 2 implementation in NENC.

Upskilling initiatives in endodontics have been published, exploring learner views and experiences.^12^^,^^20^ Similar themes were reported around training expectations, impact of upskilling on individuals and barriers to translation of teaching to clinical practice.^20^ Echoing our study findings, participants preferred clinical supervision and hands-on learning. They too reported benefits of improved confidence and personal interest, indicating personal factors are influential to workforce engagement in education and training. The most striking parallel was the perception that inadequate remuneration and integration of appropriate care pathways limited translation of teaching to service delivery.^12^^,^^20^ This supports our findings that NHS contractual arrangements and supporting infrastructure are particularly influential to engaging the workforce with Level 2 training and accreditation.

### Limitations

There are several limitations associated with this study. Survey data should be interpreted with caution due to the small sample size. Due to the sampling frame used, it was not possible to calculate a response rate, but an estimate of 6% can be made based on HEE NENC and NHS Business Services Authority data.^25^ However, these data will have an element of double counting, due to dentists working across multiple practices. The actual response rate may be somewhat higher.

To maximise responses, an opt-in distribution method was used, which may have resulted in a selection bias with motivated and interested individuals being more likely to participate. Using multiple distribution routes aimed to counteract this.

Self-perceived confidence - as our proxy of capability - is subjective and the terminology used in the Likert Scale may have had the potential to influence the nature of the response, where negativity around the term 'severely lacking confidence' may have skewed response in a more favourable direction, promoting a social-desirability bias.

It is also important to consider the influence that the interviewer (SS) and analysists (CW, SS) may have had on the qualitative data collection and analysis. However, study rigour was maintained in several ways: a topic guide was co-designed by the research team and adhered to throughout and the coding framework and data analysis were reviewed with a non-dental investigator (GV), who could minimise any bias relating to researcher role and interests.^30^

There is a need to carry out work nationally to better quantify the existing Level 2 workforce, further delineate barriers to upskilling and identify examples of successful implementation, which can be shared more widely. Given the current context of contract reform discussions and the HEE dental education reform programme, it is an opportune time to consider Level 2 funding and training models nationally.^8^^,^^31^

## Conclusion

In this region of England, the dental workforce is currently unprepared to provide Level 2 services. Although there is appetite to upskill and advantages are noted for patients and practitioners, there are multiple barriers limiting uptake. Successful implementation of Level 2 services will require collaboration of key stakeholders to provide both effective training and sustainable mechanisms that allow translation of training to viable service delivery.

## Supplementary Information

Supplementary Tables (PDF 354KB)
