# Supplementary material for: Dentists' preparedness to provide Level 2 services in the North East of England: a mixed methods study
Source: Br Dent J. 2023 Mar 7:1–8. Online ahead of print. doi: 10.1038/s41415-023-5569-3 (PMC9990555; doi:10.1038/s41415-023-5569-3)
Supplement: Supplementary file 1 — Supplementary Tables (PDF 354KB) [file 41415_2023_5569_MOESM1_ESM.pdf]

SI Table 1: Respondent self-reported confidence in a selection of Level 2 competencies

| | | Confident | | | | Neutral | | | | Unconfident | | | |
|---|---|---|---|---|---|---|---|---|---|---|---|---|---|
| Specialty Type | Specialty Specific Competency | All respondents (%) n=124 | Pre-2010 graduation respondents (%) n=61 | Post-2010 graduation respondents (%) n=63 | P value | All respondents (%) n=124 | Pre-2010 graduation respondents (%) n=61 | Post-2010 graduation respondents (%) n=63 | P value | All respondents (%) n=124 | Pre-2010 graduation respondents (%) n=61 | Post-2010 graduation respondents (%) n=63 | P value |
| **Paediatric Dentistry**<br><br>Management of patients with… | Routine oral health surveillance/ treatment where psychological development, significant anxiety, medical co-morbidity, or disability increases complexity of care delivery | 49 | 48 | 51 | NS | 31 | 34 | 27 | NS | 20 | 18 | 22 | NS |
| | Hard-tissue defects of the developing dentition | 48 | 46 | 49 | NS | 33 | 34 | 32 | NS | 19 | 20 | 19 | NS |

| | | | | | | | | | | | | | |
|---|---|---|---|---|---|---|---|---|---|---|---|---|---|
| **Periodontology** Management of patients who… | Following primary care periodontal therapy have stage II, III or IV periodontitis | 55 | 54 | 55 | NS | 24 | 16 | 32 | NS | 21 | 30 | 13 | NS |
| | Have grade C periodontitis | 35 | 38 | 32 | NS | 28 | 24 | 32 | NS | 37 | 38 | 36 | NS |
| **Oral Surgery** Management involving: | Surgical removal of buried roots and fractured or residual root fragment | 52 | 59 | 46 | NS | 20 | 18 | 22 | NS | 28 | 23 | 32 | NS |
| | Surgical removal of uncomplicated third molars involving bone removal | 33 | 34 | 32 | NS | 16 | 16 | 16 | NS | 51 | 50 | 52 | NS |
| **Prosthodontics** Management involving… | Replacement and temporisation of multiple fixed restorations | 40 | 52 | 29 | **<0.05** | 24 | 21 | 27 | NS | 36 | 27 | 44 | **<0.05** |
| | Pre-prosthetic procedures such as optimisation of abutments and occlusal adjustment | 39 | 52 | 25 | **<0.05** | 24 | 20 | 29 | NS | 37 | 28 | 46 | **<0.05** |

| | | | | | | | | | | | | | |
|---|---|---|---|---|---|---|---|---|---|---|---|---|---|
| **Special Care Dentistry**<br><br>Management of patients who have… | ASA 3 moderately controlled medical condition(s) | 40 | 38 | 41 | NS | 24 | 20 | 29 | NS | 36 | 42 | 30 | NS |
| | A disability, psychological or mental health state that means only limited examination is possible | 34 | 30 | 38 | NS | 30 | 28 | 32 | NS | 36 | 42 | 30 | NS |
| **Endodontics**<br><br>Management involving… | Anatomical challenges such as root canal curvature >30° and root length >25mm | 30 | 44 | 16 | **<0.05** | 20 | 13 | 27 | **<0.05** | 50 | 43 | 57 | NS |
| | Incomplete root development | 17 | 18 | 16 | NS | 16 | 21 | 11 | NS | 67 | 61 | 73 | NS |
| **Orthodontics**<br><br>Prescribing… | Removable appliances for the developing dentition requiring straightforward interceptive measures | 19 | 25 | 13 | NS | 12 | 18 | 6 | **<0.05** | 69 | 57 | 81 | **<0.05** |

| | | | | | | | | | | | | | |
|---|---|---|---|---|---|---|---|---|---|---|---|---|---|
| | Removable appliances in patients without skeletal discrepancies | 17 | 23 | 11 | **<0.05** | 9 | 13 | 5 | NS | 74 | 64 | 84 | NS |

NS: Not Significant; the difference is not statistically significant

SI Table 2: Respondent self-reported confidence in all Level 2 competencies by year of graduation

| | | Confident | | | | Neutral | | | | Unconfident | | | |
|---|---|---|---|---|---|---|---|---|---|---|---|---|---|
| Specialty Type | Specialty Specific Competency | All respondents (%) n=124 | Pre-2010 graduation respondents (%) n=61 | Post-2010 graduation respondents (%) n=63 | P value | All respondents (%) n=124 | Pre-2010 graduation respondents (%) n=61 | Post-2010 graduation respondents (%) n=63 | P value | All respondents (%) n=124 | Pre-2010 graduation respondents (%) n=61 | Post-2010 graduation respondents (%) n=63 | P value |
| **Paediatric Dentistry** Management of patients with… | Routine oral health surveillance/ treatment needs where psychological development, significant anxiety, medical co-morbidity, or disability increases complexity of care delivery | 49 | 48 | 51 | NS | 31 | 34 | 27 | NS | 20 | 18 | 22 | NS |
| | Hard-tissue defects of the developing dentition | 48 | 46 | 49 | NS | 33 | 34 | 32 | NS | 19 | 20 | 19 | NS |
| | Post-emergency follow-up of permanent multi-tooth injuries (including avulsion, significant luxation injuries) | 46 | 49 | 43 | NS | 19 | 18 | 19 | NS | 35 | 33 | 38 | NS |

| | | | | | | | | | | | | | |
|---|---|---|---|---|---|---|---|---|---|---|---|---|---|
| | Complicated crown fracture and uncomplicated crown/root fractures of permanent teeth | 41 | 52 | 30 | **<0.05** | 31 | 20 | 41 | **<0.05** | 28 | 28 | 29 | NS |
| **Periodontology** Management of patients who… | Following primary care periodontal therapy have stage II, III or IV periodontitis | 55 | 54 | 55 | NS | 24 | 16 | 32 | NS | 21 | 30 | 13 | NS |
| | Grade C periodontitis | 35 | 38 | 32 | NS | 28 | 24 | 32 | NS | 37 | 38 | 36 | NS |
| | Non-surgical management of gingival enlargement in collaboration with medical colleagues | 30 | 33 | 27 | NS | 21 | 20 | 22 | NS | 49 | 47 | 51 | NS |
| | Furcation defects and other complex root morphology | 28 | 33 | 24 | NS | 26 | 20 | 32 | NS | 46 | 47 | 44 | NS |
| | Peri-implant mucositis | 15 | 21 | 10 | **<0.05** | 10 | 12 | 10 | NS | 74 | 67 | 80 | NS |
| **Oral Surgery** Management involving… | Surgical removal of buried roots and fractured or residual root fragment | 52 | 59 | 46 | NS | 20 | 18 | 22 | NS | 28 | 23 | 32 | NS |

| | | | | | | | | | | | | | |
|---|---|---|---|---|---|---|---|---|---|---|---|---|---|
| | Surgical removal of uncomplicated third molars involving bone removal | 33 | 34 | 32 | NS | 16 | 16 | 16 | NS | 51 | 50 | 52 | NS |
| | Minor soft tissue surgery to remove non-suspicious lesions | 27 | 21 | 32 | NS | 15 | 17 | 14 | NS | 58 | 62 | 54 | NS |
| | Surgical removal or surgical exposure of uncomplicated ectopic teeth (including supernumerary teeth) | 15 | 20 | 11 | NS | 13 | 15 | 11 | NS | 72 | 65 | 78 | NS |
| | Surgical endodontics | 9 | 13 | 5 | NS | 12 | 17 | 8 | NS | 79 | 70 | 87 | NS |
| **Prosthodontics** Management of… | Replacement and temporisation of multiple fixed restorations | 40 | 52 | 29 | **<0.05** | 24 | 21 | 27 | NS | 36 | 27 | 44 | **<0.05** |
| | Pre-prosthetic procedures such as optimisation of abutments and occlusal adjustment | 39 | 52 | 25 | **<0.05** | 24 | 20 | 29 | NS | 37 | 28 | 46 | **<0.05** |

| | | | | | | | | | | | | | |
|---|---|---|---|---|---|---|---|---|---|---|---|---|---|
| | Occlusal reorganisation with plastic restorations, a removable appliance or both | 32 | 48 | 17 | **<0.05** | 18 | 16 | 19 | NS | 50 | 36 | 64 | **<0.05** |
| | Where aspects of occlusion need careful management to avoid premature failure of restorations | 23 | 36 | 9 | **<0.05** | 19 | 15 | 24 | **<0.05** | 58 | 49 | 67 | **<0.05** |
| | Where there are anatomical difficulties related to +/ or compromised health of soft tissues/denture bearing area | 18 | 30 | 6 | **<0.05** | 33 | 31 | 35 | NS | 49 | 39 | 59 | **<0.05** |
| **Special Care Dentistry** Management of patients who have… | ASA 3 moderately controlled medical condition(s) | 40 | 38 | 41 | NS | 24 | 20 | 29 | NS | 36 | 42 | 30 | NS |
| | A disability, psychological or mental health state that means only limited | 34 | 30 | 38 | NS | 30 | 28 | 32 | NS | 36 | 42 | 30 | NS |

| | | | | | | | | | | | | | |
|---|---|---|---|---|---|---|---|---|---|---|---|---|---|
| | examination is possible | | | | | | | | | | | | |
| | Advanced anxiety and behaviour modification techniques | 32 | 31 | 33 | NS | 25 | 25 | 26 | NS | 43 | 44 | 41 | NS |
| | Doubtful or fluctuating capacity to consent | 30 | 33 | 41 | NS | 28 | 18 | 29 | NS | 42 | 49 | 30 | **<0.05** |
| | Significant communication difficulties | 27 | 22 | 30 | NS | 23 | 30 | 18 | NS | 50 | 48 | 52 | NS |
| **Endodontics** Management involving… | Anatomical challenges such as root canal curvature >30° and root length >25mm | 30 | 44 | 16 | **<0.05** | 20 | 13 | 27 | **<0.05** | 50 | 43 | 57 | NS |
| | Incomplete root development | 17 | 18 | 16 | NS | 16 | 21 | 11 | NS | 67 | 61 | 73 | NS |
| | Location and negotiation of canals not considered negotiable in the coronal 1/3 based on radiographic and clinical evidence | 18 | 28 | 8 | **<0.05** | 25 | 23 | 27 | NS | 57 | 49 | 65 | NS |

| | | | | | | | | | | | | | |
|---|---|---|---|---|---|---|---|---|---|---|---|---|---|
| | Need for re-root treatment involving removal of well condensed root fillings | 27 | 33 | 21 | NS | 24 | 28 | 21 | NS | 49 | 39 | 58 | NS |
| | Location and negotiation of canals where another dentist has attempted but experienced problems with location, instrumentation or obturation | 16 | 20 | 13 | NS | 20 | 21 | 19 | NS | 64 | 59 | 68 | NS |
| **Orthodontics** Prescribing… | Removable appliances for the developing dentition requiring straightforward interceptive measures | 19 | 25 | 13 | NS | 12 | 18 | 6 | **<0.05** | 69 | 57 | 81 | **<0.05** |
| | Removable appliances in patients without skeletal discrepancies | 17 | 23 | 11 | **<0.05** | 9 | 13 | 5 | NS | 74 | 64 | 84 | NS |

| | | | | | | | | | | | | | |
|---|---|---|---|---|---|---|---|---|---|---|---|---|---|
| | Non-complex fixed appliance alignment in patients without skeletal discrepancies or significant anchorage demands | 15 | 25 | 5 | **<0.05** | 5 | 5 | 6 | NS | 80 | 70 | 89 | NS |

NS: Not Significant; the difference is not statistically significant
